# The epidemiological and medico-legal characteristics of violent deaths and spousal homicides through a population of women autopsied within the Forensic Medicine Department of the University Hospital of Annaba

**DOI:** 10.1186/s12905-023-02287-2

**Published:** 2023-03-25

**Authors:** Y. Mellouki, L. Sellami, L. Saker, N. Belkhadja, Y. Zerairia, F. Kaious, A. H. Mira

**Affiliations:** 1 Faculty of Medicine Badji Mokhtar, Annaba University , Route of Zaâfrania, BP205, 23000 Annaba, Algeria; 2 Service of Forensic Medicine Hospital and University Center of Annaba. Route of Strasbourg, 23000 Annaba, Algeria

**Keywords:** Violent death, Female victims, Spousal homicide, Autopsy

## Abstract

**Background:**

Domestic violence is a real public health problem with considerable consequences, ranging from minor injuries to death. Our study aims to determine the epidemiological and forensic characteristics relating to the violent mortality of women, and more particularly spousal homicide.

**Methods:**

To do this, a double survey was conducted. The first step was descriptive and retrospective, and the second survey was analytical and prospective. This latter step covered the most populous age group of murdered women in Algeria, which is eighteen-year-old and over, and subjected a number of these female victims to a medico-judicial autopsy at the level of the thanatology unit for over four years counting two years for each survey (2017–2018 and 2019–2020). Data were entered and processed using Epi-info6 software.

**Results:**

During the initial period of our study, we identified 35 cases of violent deaths involving women and representing a frequency of 5.71% of the thanatological activity.

During the second period, 12 spousal homicides were recorded and autopsied, representing a frequency of 1.79% of all forensic deaths in the corresponding study period.

The average age of the victims was evaluated at 33 ± 12.91 years, with extremes of 19 to 56 years. The age of the perpetrators of spousal homicide was evaluated at 42 ± 10.76 years with extremes ranging from 30 to 60 years.

For victims of violent death and spousal homicide, inactivity was a strongly implicated risk factor, with respective frequencies of (88.57%) and (58.33%). Two-thirds of the persecuted women were completely unknown to the healthcare environment and had never consulted a medical professional. This parameter could be one of the predictive signs of spousal homicide.

The marital home was the preferred location for violent deaths and spousal homicides. These crimes occurred variably during the period of marriage and eventually after divorce.

As for the modus operandi, the perpetrators use many sharp and spinous weapons, including firearms and blunt objects.

**Conclusion:**

Autopsy and medico-legal investigations took a decisive interest in the identification of the causes of spousal homicide; indeed, many serious traumatic lesions incompatible with life have been highlighted. We underline the crucial role that healthcare professionals must play in the process of identifying and evaluating potentially risky situations.

## Background

The mortality of women, and more particularly that which occurs as a result of domestic violence, represents a real public health problem with lethal consequences. The international literature on spousal homicide is poor and the references are limited. Nevertheless, some studies have attempted to draw up the profile of the victims or that of the perpetrators, especially from a criminological and/or psychopathological point of view.

According to the latest estimates from the World Health Organization (WHO), the intimate partner is the perpetrator of the homicide in approximately 38% of homicides committed against women [1].

According to official statistics from the Government of Canada, during the year of 2003, seventy-eight (78) people were killed by a spouse, including 64 women and 14 men. This homicide rate fell slightly in 2003 compared to 2002, when 83 victims were counted [2].

In Italy, the incidence of femicides is increasing more and more in recent years and it accounted for 30.9% of all homicides in 2011 [3].

An Italian study assessed the characteristics of female homicides and femicides based on autopsy reports of female homicides that occurred in the judicial district of Bologna, over a period of 70 years, on a sample of 172 female homicides, including 103 femicides. This study concluded that there was a statistically significant association between homicide and the victim-offender relationship and that over time the main age of victims increased [4].

Another study from Italy examined all cases of female homicides using autopsy reports at the Institute of Forensic Medicine in Milan over twenty years (1999–2019).

Two hundred (200) murders of women were noted, among more than 15,000 autopsies performed, and 535 homicides were recorded at the Institute of Forensic Medicine in Milan over the period considered, with an average of 9.5 femicides per year [5].

A Turkish study, in Eskişehir province, investigated the cases of women killed by their partners (husband, boyfriend, ex-husband, or ex-boyfriend) over 25 years (1992 and 2016), through the data collected from autopsy and toxicology reports. According to the results of this study, 148 women were victims of femicide over 25 years, and the number of cases continues to increase every year [6].

Another Turkish study was conducted in Istanbul, on a sample of 20,486 forensic autopsies carried out for five years (2006, 2010). Of these deaths, 537 were related to female violence and 12.9% of female deaths were due to intimate partner violence, which accounted for 2.6% of all autopsies performed [7].

A third Turkish study accounted 162 cases of femicides that occurred in 12 cities in Turkey over ten years (January 1st, 2000, to December 31st, 2010). According to this study, 80 women were killed by their intimate partner, and 81 women were killed by one of their relatives (friends or sexual partners) [8].

In the United Kingdom, 37% of all women were murdered by their current or former intimate partner compared to 6% of men [9].

This very question of spousal homicide arose the interest of the team of the medico-legal institute of Paris, which tried to highlight the seriousness of this phenomenon. A study was conducted to evaluate the frequency of spousal homicides on a representative sample of 652 deaths over a period of seven years. This study showed that 31% of female homicides were committed by intimate partners, 20% by sexual partners, and 15% by individuals unknown to the victims [10].

A French team from the Forensic Institute of Tours was also interested in this phenomenon of spousal homicide and carried out a study for four years (2000 -2003). This study analyzed two years of autopsies of spousal homicides and showed that frequently there was a link between the victim and the perpetrator of the homicide. Indeed, in 50% of homicides, the victims knew their assailants; in 25% of cases, the aggressor was a family member, and in 17% of the cases, it was spousal homicide [11].

In France, a frequency of 10% of male deaths and 7% of female deaths were attributed exclusively to violent acts. Violence occupies the third etiology of mortality after cardio-circulatory diseases and tumors. Domestic violence is considered to be one of the main causes of female mortality [12].

The World Health Organization (WHO) recently released its report on violence-related mortality data. No less than 38% of women who were murdered were killed by their intimate partners, and 42% of these women received physical injuries or sexual violence from a partner [13].

It is important to note that we do not have enough data on spousal homicides in Algeria and its neighboring countries. The objective of this study is to determine the epidemiological and medico-legal characteristics of the violent deaths of women, and more particularly, of spousal homicide.

## Material and methods

This study was based on a double survey, initially, retrospective, for two years (2017, 2018) and secondarily perspective, also for two years (2019, 2020), relating respectively to the violent mortality of women and intimate partner homicide. The initial, retrospective, survey focused on women who died in a violent context without considering the circumstances of the occurrence and regardless of the medico-legal forms, during the period between January 1st, 2017, and December 31st, 2018. The second, prospective, survey concerned exclusively women who died as a result of violence caused by an intimate partner and who underwent a forensic autopsy during the period between January 1st, 2019, and December 31st, 2020.

During this double survey, all women aged 18 and over (the most prominent age group in Algeria) who died in a context of violence and on whom a medico-judicial autopsy was performed, were included.

Our investigation took place within the thanatology unit of the Forensic Medicine department at IBN ROCHD Hospital and University Center, taking charge of autopsy activities and post-mortem explorations while seeking justice.

Our protocol included a detailed external examination to determine a complete lesion assessment by identifying the type of lesions, and their locations, and evoking the vulnerable instruments used to cause these injuries, as well as the medico-legal reasoning in terms of imputations and causes of death.

The internal examination made it possible to highlight the complete lesion assessment of the organs and viscera affected, as well as the fatal injuries (number, depth, and cause of death).

Our protocol ends with toxicological and histological examinations according to the medico-legal circumstances and the legal requirements.

For the data collection of our study (sociodemographic parameters, forensic characteristics, and data related to the circumstances of death), we based investigation on the questioning of the women's relatives and those around them, reports from the police and national gendarmerie services, as well as judicial files carried out by the competent court. The data obtained were processed using the Epi info6 software.

All measures and provisions related to the principles applied to the ethics of research on human beings, namely the confidentiality of the information obtained and the informed consent of the families and beneficiaries of the participants in the double survey, were rigorously respected and in full compliance with the Helsinki Biomedical Research Ethics Guide).

The Ethics and Research Committee in Health Sciences of the IBN ROCHD University Hospital gave a favorable opinion to this double survey on the violent mortality of women and spousal homicide by approving the research protocol and emphasizing the confidentiality of the collected data.

Sheets in the triple language (Arabic, French, and English), explaining the objectives and framework of the study, were prepared beforehand and presented to families, relatives, and beneficiaries before each collection of information.

## Results

### The retrospective survey

During the two years of the retrospective study (2017 and 2018), women who underwent a forensic autopsy for reasons of violent death represented a frequency of (5.71%): 35 women victims of violent death out of a total number of 570 autopsies performed.

**Fig. 1:**
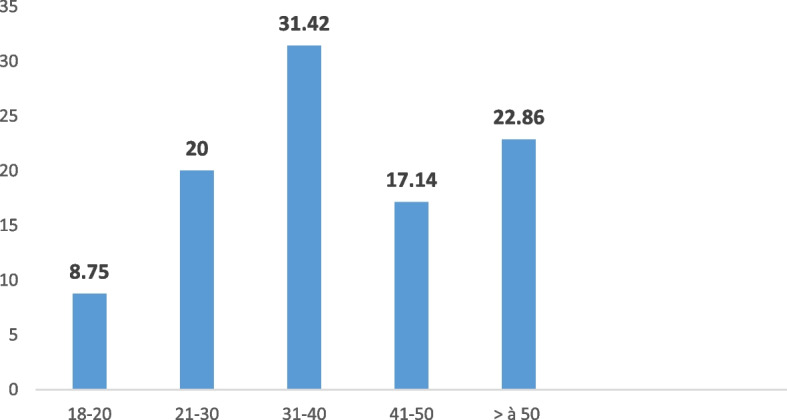
Age groups of women’s victims of violent deaths

All age groups were concerned, but to a greater extent young women, particularly those aged between 31 and 40 (31.42%). Figure 1 shows the respective frequencies of the age groups.

Married women were the most affected. Indeed, nearly three-quarters of the victims are legally married (71.42%).

The remaining proportion was shared between single women (14.28%), widowed women (11.42%), and divorced women (2.89%).

The overwhelming majority of female victims of violent death (93%) were inactive at the time of the murderous act.

Eighty-eight point fifty-seven percent of them had no profession, and 5.71% were retired.

**Fig. 2:**
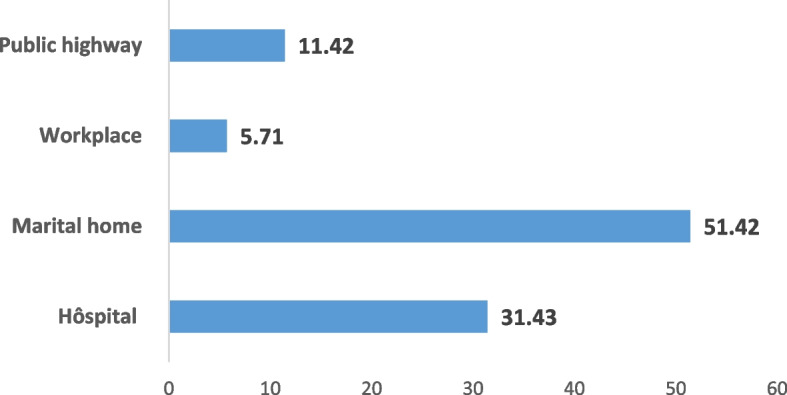
Sites of occurrence of the violent death of women

A frequency of 82.85% died in the marital home and at the hospital following complications from the initial trauma. Figure 2 lists the sites where violent deaths occur among women.

**Fig. 3:**
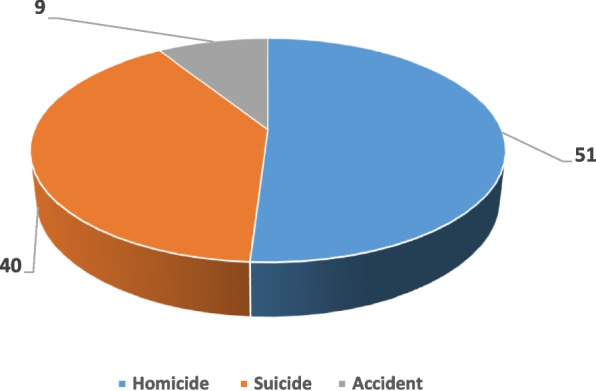
Forensic forms of violent deaths

Homicide is the most represented forensic form, with a frequency of 51.42%, followed by suicide with a frequency of around 40%. Figure 3 shows the respective frequencies of the three forensic forms of violent female death.

**Fig. 4:**
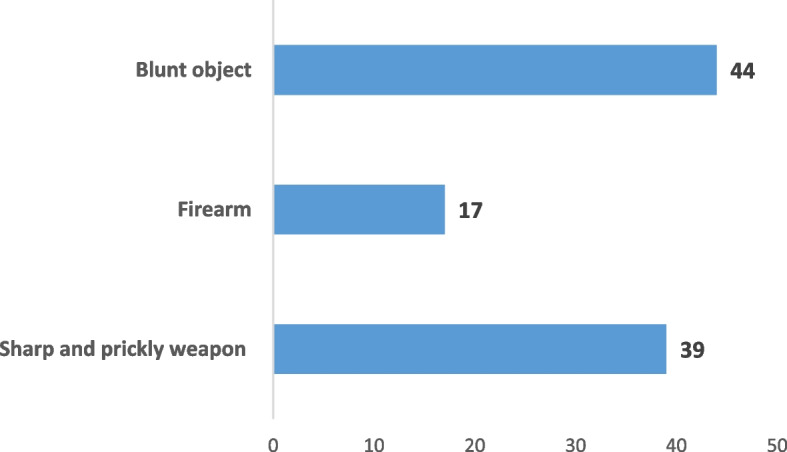
modus operandi in female homicides

In terms of injury tools of homicide victims, blows with blunt objects represent the most frequent operative mechanism (44.44%), followed by injuries caused by sharp instruments (39%) while the use of firearms was relatively lower (17%) (Fig. 4).

**Fig. 5:**
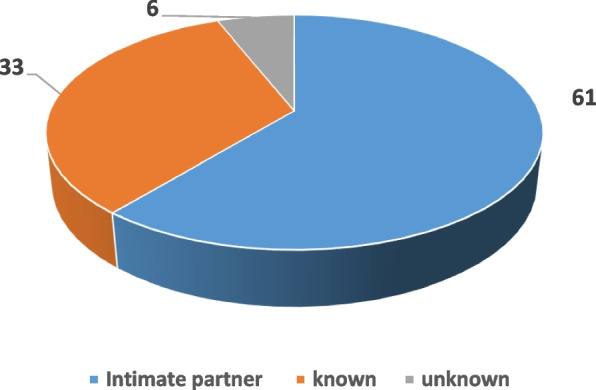
Presumed perpetrators of intentional homicides *N* = 18

During suicides, hanging is the mostly used method by the victims (42.85%). However, the use of other processes, namely jumping off a high place (28.57%) was added to the ingestion of caustic products (21.42%) and drugs (7.14%). In homicides, the spouse is the perpetrator in 61.11% of cases. The perpetrator is unknown to the victim in 33.33% of the cases. Figure 5 shows the link between the perpetrator and their victims.

**Fig. 6:**
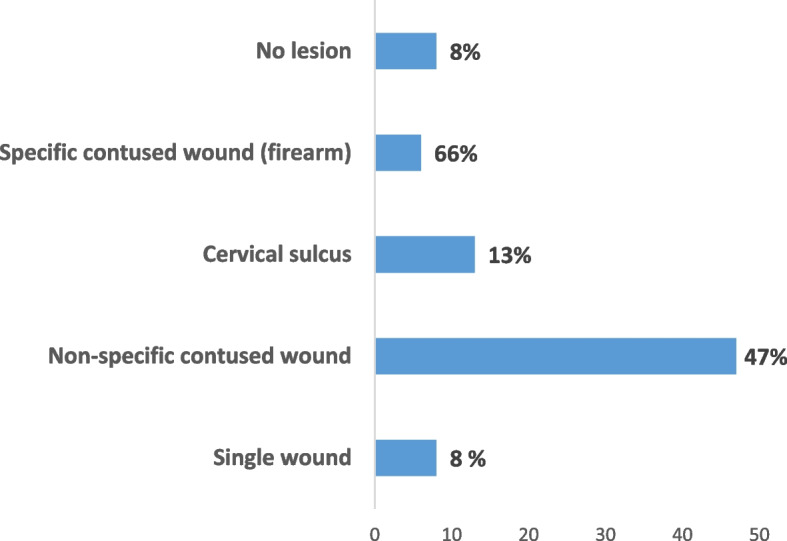
Traumatic assessment on external examination of victims (No. 35)

Forensic examination showed the following frequencies: Non-specific blunt wounds (47%), simple wounds (26%), and blunt gunshot wounds (6%) (Fig. 6).

**Fig. 7:**
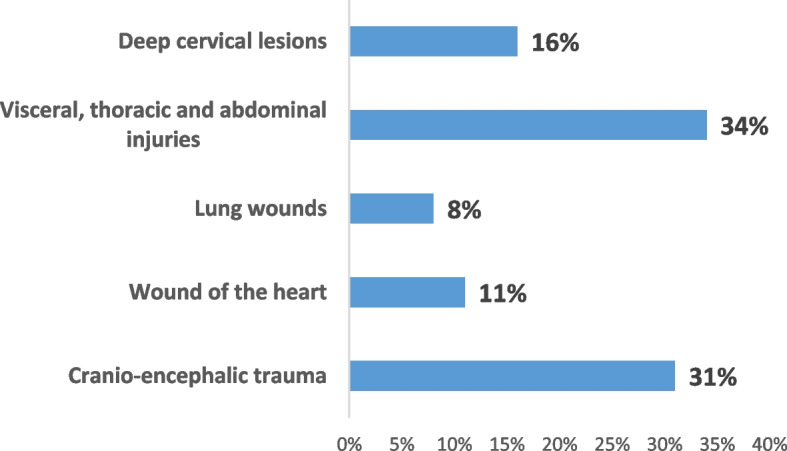
Various internal violence lesions revealed by the forensic autopsy

Thoracic and abdominal lesions were the most frequently observed injuries in victims (34%). Figure 7 shows the different lesions of internal violence highlighted by the forensic autopsy.

**Fig. 8:**
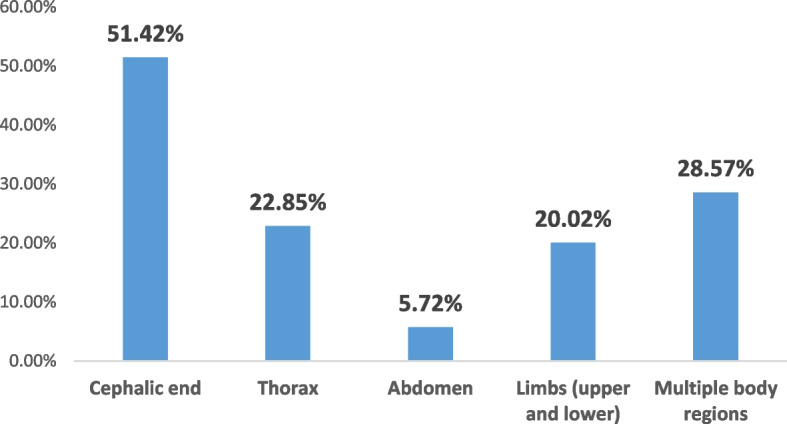
Lesion topography of fatal trauma in autopsied women

The cephalic extremity was the region of predilection for this type of fatal trauma (51.42%), followed by the category **“**multiple anatomical regions of the body**”** where several body segments were affected (28.57%). The other traumatic locations were indicated in detail in Fig. 8.

The use of complementary investigations represented an essential tool in the identification of the violent causes of death and in particular those secondary to intoxication. However, the results remain highly dependent on several parameters (date of the autopsy, date of sampling, nature of the sample and its storage, cause of death, etc.). Toxicological research was almost systematic among victims who died following a homicide: (N: 14/18: 77.77%) of all homicides and (N14/35: 40%) of all victims of violent deaths.

The recourse to histological examination was rare. Thus, according to our results, we note the following frequencies: (N: 6/18), a frequency of 33.33% of all homicides, and (N: 6 /35), representing a frequency of 17.14% of all violent deaths. Table 1 illustrates the different examinations carried out during autopsies of victims of violent deaths.

**Table 1:** Thanatological investigations carried out on victims of violent deaths

**Violent Death**	**Toxicology**		**Histology**	
	**Number**	**Frequency (%)**	**Number**	**Frequency(%)**
Homicide (n: 18)	14	40	06	17,14
Suicide (n: 14)	10	28,57	00	00
Accident (n: 03)	00	00	00	00
**Not examen**	11	31,42	29	82,58
Total	35	100	35	100

### The prospective survey

During the prospective survey (2019—2020), 12 spousal homicides were reported out of a total number of 670 forensic autopsies carried out during the aforementioned period, a frequency of nearly two percent (1.79%) of all medical-legal deaths. The sample studied had an average age of 33 ± 12.91 years, with extremes of 19 to 56 years. The age group between 21 and 30 is the most represented one, with a frequency of (50, 33%), followed by those aged over 50 years old, with a frequency of (25%).

**Fig. 9:**
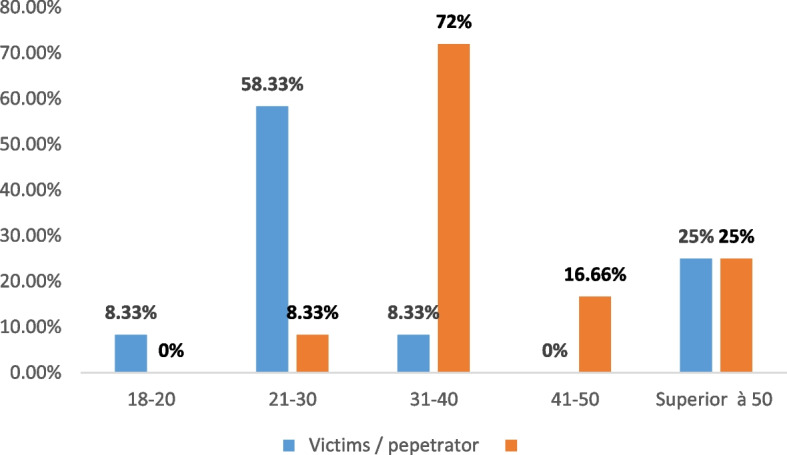
Age of victims and perpetrators of spousal homicide

The perpetrators of spousal homicides had an average age slightly higher than that of the victims: about 42 ± 10.73 years with extremes ranging from 30 to 60 years old. Three-quarters (72%) of the perpetrators were between 31 and 40 years old and 25% were over 50 years old. Two-thirds of the victims in our sample are married (66.66%), while one-third maintain an unofficial relationship (sexual partner) (Fig. 9).

The description of the Socio-demographic characteristics of victims and perpetrators of spousal homicide is detailed in Table 2.

**Table 2:** Socio-demographic characteristics of victims and perpetrators of spousal homicide

**Marital status**	**Number *N* = 12**	**Percentage**
• Friend	4	33.3%
• Bride	8	66.7%
Victim's age (years)		
18–20	1	8.3%
21–30	7	58.3%
31–40	1	8.3%
41–50	00	00
51–60	3	25.0%
• Means (standard deviation)	33.00 ± 12.91	
• 95% CI for the means	24.79 à 41.20	
• Extremes	19–56	
Perpetrator's age		
30–40 ans	7	58.33%
41-50ans	2	16.7%
51–60 ans	2	16.7%
60 et +	1	8.3%
• Mean (standard deviation)	42.00 ± 10.73	
• 95% CI for the mean	35.16 à 48.83	
• Extremes	30–60	
Professional status of the victim		
Student	4	33.33%
Public service	1	8.33%
No occupation	7	58.33%
Professional status of the perpetrators		
Free fonction	4	33.3%
Public service	4	33.3%
Factotum	2	16.7%
Unemployed person	2	16.7%
Place of Death		
Marital home	5	41.7%
Hospital	4	33.33%
Public place	2	16.7%
Workplace	1	8.3%

Only one victim worked full-time. More than half of the victims (58.33%) were unemployed unlike the rest of the victims (33.33%) who were students. Contrary to the professional status of the victims, that of the perpetrators was composed in a proportion of 84% of employees and civil servants, whereas for the perpetrators, only two of them were unemployed at the time of the family tragedy.

In terms of the circumstances of death, our study evoked above all the notion of misunderstanding and argument which ended in tragedy (66.66%), financial reasons (16.66%), and beatings without the intention of causing death (8 0.33%); however, in rare cases, no reason has been reported. A third of the victims (33.33%) were known to the healthcare environment and consulted for injuries and the need for medical care. Eight victims were unknown to the health services.

Two women consulted at the forensic medicine unit of the legal medicine department, one at the gynecology and obstetrics department, and the other at the surgical emergency department for physical violence caused by an intimate partner.

Regarding the period of the potential risk of violent acts, except in cases where the information relating to this parameter was missing (33.33%), a spousal homicide occurred much more during the period of marriage than divorce. Respective frequencies of 33.33% and 25% were noted, while temporary separation was mentioned only rarely (8.33%).

The site of death was the marital home (41.66%); the second was the hospital (33.33%). For two victims, death took place in a public space.

In terms of modus operandi or mechanisms of death, these were multiple and shared equally between three mechanisms: wounds by bladed weapons, wounds by projectiles from firearms, and blows by a blunt object.

The medico-legal examination revealed a varied and characteristic external assessment, consisting mainly of non-specific contused wounds, characteristically sharp and stinging weapon wounds, and finally particular contused wounds caused by firearm projectiles.

**Fig. 10:**
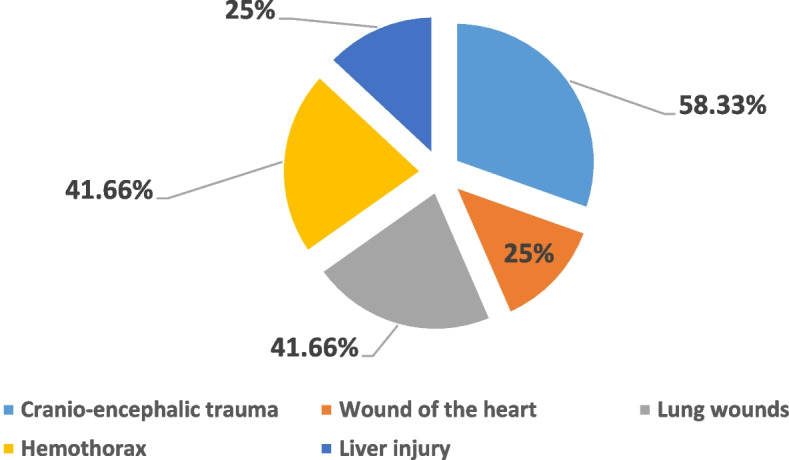
Internal injury report in spousal homicide

The internal assessment was mainly characterized by traumatic craniocerebral lesions (58.33%). The other fatal lesions were represented in Fig. 10.

Toxicological investigations showed the presence of alcohol in a single victim and cannabis in two victims. However, they came back negative in the rest of the victims (*n* = 09).

The cause of death in spousal homicide is mainly represented by hemorrhagic shock (50%), followed by craniocerebral trauma (33.33%).

## Discussion

Violence against women is frequent; it is a source of multiple consequences ranging from simple trauma to death. It is difficult at our level to know the exact number of homicides resulting from domestic violence.

During the initial investigation, we identified 35 cases of violent death involving women, representing a frequency of 5.71% of all medico-legal deaths; whereas, during the prospective cross-sectional survey 12 spousal homicides were recorded out of some 670 forensic autopsies, representing a frequency of 1.79% of all forensic deaths.

As an indication and according to the results of a systematic review conducted by a research team, under the direction of the World Health Organization during the year 2013, a frequency of approximately 13.5% of homicides was committed by the intimate female partner and this frequency is six times higher than that of homicides against male partners [14].

Young victims were more affected by this form of violent death: respective frequencies of 31.42% and 50, 33% were noted for victims aged between 31 and 40 years old during the retrospective survey and 21 and 30 years old during the prospective survey.

According to the Italian study described earlier in this article, 50% of the victims were between 18 and 49 years old, and the spousal homicides were committed in the domestic context (78.5%) by male perpetrators (85%), having a relationship with the victims, as intimate or ex-intimate partners (73.5%) [5]. While for the Turkish study, female victims of spousal homicides had an age range between 19 to 29 (38.5%), and (15.5%) of them were 18 or younger [6].

The average age of the victims was evaluated at 33 ± 12.91 years, with extremes of 19 to 56 years.

Regarding this criterion, a retrospective analysis of forensic autopsy records of adult femicide victims in Taiwan over ten years showed that the average age of victims killed by intimate partners (40.0 years) was younger than those killed by non-intimate partners (48.6 years) [15].

This result concerns us directly and requires objective answers because this observation is completely contrary to other series where the average age of the victims was relatively higher (average age 45.8 with extremes of 18 and 85 years [16].

According to our study, elderly victims are remarkably less affected. For the Turkish series, the medium age of victims was 43 years old and a significant proportion of victims (49.7%) were between 21 and 40 years old [7].

The average age of perpetrators of spousal homicides is 42 ± 10.73 years with extremes ranging from 30 to 60 years old.

Three-quarters of the perpetrators were between 31 and 40 years old and 25% are over 50 years old.

This rate joins that of the Canadian series [17]and other series of the European continent [9] where an average age of 43.2 years was noted and it is relatively high compared to the Tunisian series, where this rate was 35, 2 years [16].

A clear predominance of married women with respective frequencies of (71.42%) and (66.66%). This frequency may be superimposed on that noted by the various series among living victims where domestic violence is widespread [18].

A remarkably high frequency of unemployed victims was recorded, both by the retrospective survey (88.57%) and by the prospective survey (58.33%). This confirms the frequent implication of socio-economic conditions where the share of unemployment and inactivity were frequently reported.

The presence of previous violent episodes could be considered an early warning sign of spousal homicide.

Unlike the two Canadian [17] and Parisian [19] series, our survey showed a frequency of more than half (56.3%( of victims who are known to the care services and who have suffered previous domestic violence. This frequency is comparable to the Tunisian series [16].

For our study, 12.5% of the authors had a history of staying in a psychiatric environment, this result is in disagreement with the Tunisian series, where 80% of the authors suffered from a mental disorder [16].

This highlights the importance of a systematic screening of situations of domestic violence at the level of basic care units and the possibility of alarming the victims who present serious injuries which could end in a family tragedy.

The marital home represents the primary site of predilection for violent deaths and marital homicides. The hospital comes in the second position where death occurs following the various complications of the initial domestic injuries. This observation is shared with a French study [20].

In terms of the period of cohabitation during which the death took place. It appears that spousal homicide occurs much more during marriage than divorce, contrary to the results of the two series above [16, 19]; nearly a third (31.3%) of the same proportion of perpetrators were in a separated couple, and more than a third (37.5%) were in the process of separation at the time of the murderous act.

According to the results of the retrospective survey, in more than half of the cases (51.42%), the violent deaths are the result of a criminal violent death while in 40% of the cases the victims presented injuries similar to those observed in suicides.

The results of an American study in North Carolina analyzing the precursors to female suicides suggested that intimate partner violence was a precursor in at least 4.5% of single suicides and intimate partner violence was present in 6.1% of suicides overall [21].

The modus operandi in homicide was represented in almost half of the cases by blows with blunt objects and bladed weapons.

Firearms are rarely used, contrary to the results of a French series where 70% of murders were committed with a firearm [22].

In spousal homicides, perpetrators appear to use as many sharp and pointed weapons as firearms and blunt objects. For the Canadian series, 62.5% used a firearm and 43.8% of the perpetrators of spousal homicides resorted to excessive violence [19]. While according to the Italian study, the homicides were mainly perpetrated, with sharp and prickly instruments (32%), blunt instruments (21.5%), gunshots (18.5%), and asphyxiation (16.5%) [5].

As for the mechanism of death, in the Turkish study, spousal homicides were most often committed with a sharp object (49.3%) [6].

For the Istanbul City Series, violent deaths were committed by the following mechanisms: Gunshot wounds (50.1%), strangulation (8.4%), and stabbing wounds (28.3%) [7].

According to another Italian study conducted on a sample of 319 cases of spousal homicide, sharp and spinous weapons were used in (31.97%), firearms in (27.27%), and asphyxiation in (16.30%) of the cases. These homicides were committed inside the marital home in (65.51%) of the cases [23].

In deaths by suicide, we have found that the most common means used is hanging due to its radical nature, in addition to other means namely precipitation from a high place, ingestion of caustic products, and drug intoxication.

In homicides, the alleged perpetrators are represented in nearly two-thirds (61.11%) of the cases by the spouse. This immediately raises the urgency of a systematic screening of situations of intra-family violence and in particular violence provoked by the intimate partner.

In terms of motivations for spousal homicide. This study evoked above all the notion of disagreement and dispute which leads to a dramatic tragedy in addition to financial motives and blows without the intention to cause death, contrary to the Canadian series [17] where 43.8% of the perpetrators report respectively, and in decreasing order of frequency: marital separation (18.8%), mental disorders (6.3%), and pecuniary motivations. For the Parisian series [19], the reasons reported were respectively the perpetuation of violence (94%), separation (16%), and verbal threats of death or suicide by the perpetrators (12%).

For the Turkish series, the perpetrators declared, during their trials, before the court that the most frequent reason for homicide was the request for divorce or separation (61.5%) [6].

According to a study conducted on homicide-suicide in the province of KONYA in Turkey, out of ten cases of homicide-suicides, the precipitating reason for this act was an imminent divorce in four cases, a depressive syndrome in two cases, an antisocial personality disorder in one case, and a pedophile suffering from reactive depression [24].

The results of a study conducted on a sample of 86 cases of femicide suggest that it was perpetrated disproportionately by intimate partners (current or past) rather than by strangers with high levels of litigation and conflict representing significant precursors to spousal homicide [25].

The external examination revealed traumatic bodily injuries in almost all of the abused victims. Their nature depends closely on the instruments used: non-specific contused wounds, located mainly at the level of the cephalic extremity, and simple wounds caused by a stab wound in a third of the victims, with their distribution over several segments of the body.

Signs of passive constriction of the neck, in the form of a cervical hanging furrow, are not insignificant while blunt force injuries characteristic of gunshot wounds have been noted in one-third of domestic homicide victims.

The medico-legal autopsy and the complementary investigations are of fundamental interest in the knowledge of the causes of violent death and particularly in marital homicide; various and multiple traumatic lesions were highlighted by the autopsy.

These are mainly traumatic cranio-encephalic lesions, thoracoabdominal visceral lesions, and, to a lesser extent, deep cervical lesions. For the Jordanian study, the most common cause of death was gunshot wounds, and the severity of the violence was judged by the highest number of serious injuries [26].

Heart wounds were noted in one victim of violent death, in 10, and a quarter of victims of spousal homicide.

Toxicological research is systematic in violent deaths by intentional homicide and suicide, given its involvement in medico-legal circumstances, whereas it is less often carried out in the event of accidental death.

The histological examination is of less utility compared to the toxicological examinations in the establishment of violent causes of death. Finally, in spousal homicide, the cause of death is essentially due to hemorrhagic shock or complications of traumatic brain injury.

## The strengths and weakness

The strengths of this survey are the dual nature of the survey, (retrospective and prospective), the methodology and source of study data (autopsy reports), and its duration extending over four years.

The limits of our study are the cross-sectional survey sample which is small despite the two-year duration. Some parameters could be incomplete because the lifting of bodies is not systematic and remains dependent on the judicial decision. Some data related to the context of the death may lack precision as it was collected from the entourage and police reports during the preliminary investigation.

## Conclusion

This study highlights the reality of violent deaths against women in the shape of spousal homicides, which were hidden despite social, cultural, and legal charges. The real figures remain difficult to grasp due to the underestimation and the underreporting of family violence and violence caused by intimate partners for social, cultural, and religious reasons, etc.

Particular attention on the part of professionals working in the field of forensic medicine is perfectly justified and essential in the reception and care services for abused women in the forensic investigation units. Young married women without professional activity represent the most vulnerable category of homicide victims.

While in terms of predictive signs, our study identified two main factors, which are the place of occurrence of the violent death, represented by the marital home, and the period of marriage.

For the mechanism of death in homicide, our study identified the three classically used modes namely blows by blunt objects, wounds by sharp and prickly weapons, and finally wounds by firearms.

Three main factors have been identified as motives for committing the criminal act: the notion of argument and disagreement, financial motives and difficulties encountered by couples, and recurring altercations.

We underline the interest in screening for domestic violence as well as the search for risk factors during the management of violence induced by the intimate partner; this could increase the probability for the actors of the management (police, social and judicial forces) to anticipate the murder before its occurrence and thus saving the women from irreparable harm.

This study indicates that forensic medicine services could be a reliable source of statistical data and confirms that forensic research could help identify predictors of domestic homicides, which absolutely, must be taken into account in strategies to combat domestic violence and in the design of support systems and prevention strategies in the fight against domestic violence.

The results of this work must be pursued by other large-scale studies and bigger samples to have more solid scientific knowledge allowing the establishment of strategies, aimed at evaluating the potential risk of conjugal homicide at an early stage and providing appropriate support to victims on time.

## Data Availability

Datasets generated and/or analyzed during the current study are not publicly available due to the investigative secrecy of forensic autopsy report findings but are available from the corresponding author at reasonable request. Sheets explaining the objectives and framework of the study were prepared beforehand and presented to the families of rights holders before each collection of information.
